# Temporal Variability in the Spiking Activity of Neurons in the External Globus Pallidus in Healthy and Parkinsonian Monkeys

**DOI:** 10.1523/JNEUROSCI.1283-25.2026

**Published:** 2026-03-02

**Authors:** Adriana Galvan, Xing Hu, Anne-Caroline Martel, Kate S. Heffernan, Annaelle Devergnas, Thomas Wichmann

**Affiliations:** ^1^Emory National Primate Research Center, Emory University, Atlanta, Georgia 30329; ^2^Udall Center of Excellence for Parkinson’s Disease Research, Emory University, Atlanta, Georgia 30322; ^3^Department of Neurology, School of Medicine, Emory University, Atlanta, Georgia 30322

**Keywords:** basal ganglia, electrophysiology, MPTP, Parkinson's disease, rhesus macaque

## Abstract

The external segment of the globus pallidus (GPe) is a critical node within the indirect pathway of the basal ganglia. In vivo recordings in primates and other animals have shown that most GPe neurons exhibit high-frequency firing with pauses, whereas others display low-frequency firing with frequent bursts. Although these two firing patterns have been considered to represent distinct neuronal subtypes, evidence suggests that they may instead reflect extremes of a continuous spectrum of firing patterns and rates within this structure. Using long-duration electrophysiological single-unit recordings (5–60 min) in awake rhesus macaques (4 females, 3 males), we found that the firing rates and patterns of individual GPe neurons vary substantially over time. In parkinsonian monkeys, GPe neurons continued to show significant variability in firing, although within a more constrained range. Given the temporal fluctuations, long recording durations are necessary to accurately represent the range of firing characteristics of GPe neurons, both in healthy and parkinsonian states. Most GPe cells displayed, at different time points, characteristics of high-frequency or low-frequency firing modes, but for most of the time fired in an undetermined mode. We conclude that the firing of GPe neurons transition along the extremes of a continuous spectrum of properties, and therefore GPe cells cannot be classified based solely on firing patterns in in vivo recordings.

## Significance Statement

The external globus pallidus (GPe) is a key modulator of basal ganglia activity, influencing movement in health and disease. Classic studies in monkeys categorized GPe neurons into two main types based on their firing patterns. We show that most GPe neurons exhibit substantial variability in firing over minutes, calling into question whether firing patterns alone can reliably define cell types. In parkinsonian monkeys, the firing variability persists. These findings underscore the need for long-duration recordings to capture the dynamic nature of GPe activity and suggest that disease states may constrain this variability, offering new insight into the alteration of the activity of basal ganglia neurons in the parkinsonian state.

## Introduction

The first report of in vivo extracellular recordings in the external segment of the globus pallidus (GPe) of awake monkeys described that most GPe neurons show high-frequency spiking activity, interspersed with pauses (high-frequency discharge with pauses, HFD-P), while a smaller proportion was said to show low-frequency discharge with bursts (LFD-B; DeLong, 1971). Similar patterns of pallidal discharge have been demonstrated by other authors, both in primates (Katabi et al., 2022) and rodents (Bugaysen et al., 2010; Benhamou et al., 2012).

There is evidence, however, that the HFD-P and LFD-B subtypes of GPe neurons are only the most recognizable extremes of a continuous spectrum of properties of GPe neurons. This view is supported by in vivo and in vitro recordings in rodents which found that the firing properties of the population of GPe neurons distribute along a continuum, with specific cells firing within more limited boundaries of firing rates and patterns (Abdi et al., 2015; Cui et al., 2021). Furthermore, observations in rodents showed that GPe neurons display a wide range of firing rates and patterns (Deister et al., 2013). The firing pattern heterogeneity in in vivo recordings may arise, at least in part, due to shifts in firing behavior of the same neurons, as has been reported in rodent studies (Deister et al., 2013). Such variations in firing patterns may only be detectable when recordings extend over long time periods (Elias et al., 2008).

The loss of nigrostriatal dopamine fibers associated with parkinsonism induces multiple alterations in GPe, where neuronal firing becomes slower, may show more frequent bursts, and becomes more synchronized (Galvan et al., 2015; Courtney et al., 2023). However, the stability of firing patterns of GPe neurons in the parkinsonian state has not yet been investigated.

In this study, we took advantage of the availability of long-duration electrophysiological recordings of the spontaneous activity of rhesus macaque GPe neurons to analyze the stability of the firing patterns of these cells in the healthy and parkinsonian states [induced by injections of the neurotoxin 1-methyl-4-phenyl-1,2,3,6-tetrahydropyridine (MPTP)]. Our findings suggest that, given substantial fluctuations in the individual firing of GPe neurons, these cells cannot be classified solely on their firing characteristics and that long recording sessions are necessary to accurately describe GPe activities in healthy and parkinsonian conditions.

## Materials and Methods

### Animals

Seven adult rhesus macaques (*Macaca mulatta*; 4 females, 3 males, ages 4–7 years at the start of the experiments) from the Emory National Primate Research Center colony were used in this study (Table 1). All procedures were approved by the Animal Care and Use Committee of Emory University and performed in accordance with the Guide for the Care and Use of Laboratory Animals (NRC, 2011) and the US Public Health Service Policy on the Humane Care and Use of Laboratory Animals (revised 2015).

**Table 1.** Characteristics of animals in this study

Animal	Sex	Age at start of study (years)	Total MPTP dose (mg/kg)	Duration of MPTP treatment (days)
332	Male	5.16	N/A	N/A
353	Male	5.25	N/A	N/A
342	Female	5.05	15.1	266
354	Female	6.30	18.4	315
350	Female	7.09	8	182
319	Female	4.44	2.1	112
221	Female	4.25	6.05	172

The table describes the sex, age and, for animals receiving MPTP, the total dose, and duration of treatment.

### Recording chamber placement

After being trained to sit in a primate chair (McMillan et al., 2014), the animals underwent a surgical procedure for placement of chronic recording chambers and a head-holding bolt on the animal’s skull. They were sedated with ketamine (10 mg/kg), intubated, and then maintained on inhalation anesthesia with isoflurane (1–3%). The animal's vital signs were monitored throughout the surgery (heart rate, respiration, oxygen saturation, and end-tidal pCO_2_). The animals were placed in a stereotactic frame. Two trepanation holes were made in the skull using a trephine, and stainless-steel recording chambers were placed over the holes (Crist Instrument; inner chamber diameter, 16 mm). The chambers were stereotaxically aimed at GPe and embedded into an acrylic skull cap, along with a stainless-steel head-holding bolt and bone screws. In three of the animals, we also implanted epidural screw (diameter: 0.25 mm, length 0.4 mm) on each side of the skull through small burr holes over the primary motor cortex or premotor cortices which were connected to a serial connector. These screws served as electrocorticogram (ECoG) recording electrodes.

To manage pain and prevent infection, the animals were treated perioperatively with ceftriaxone (25 mg/kg daily for 5 d), banamine (1 mg/kg four times daily for 3 d), and buprenorphine (0.01 mg/kg four times daily for 2 d). These treatments started on the day of the surgery, before the start of the procedure.

### Electrophysiological recordings

During all electrophysiological recording sessions, the monkeys sat on a primate chair with their heads restrained and were continuously monitored with live video. They remained awake throughout the recording sessions, as judged by observations of eye opening and body movements. Standard tungsten microelectrodes (*Z* = 0.5–1.0 MΩ at 1 kHz; FHC) were lowered into the GPe with a microdrive (NAN Instruments or MO-95B microdrive, Narishige). Extracellular neuronal signals were amplified (DAM-80 amplifier; WPI) and filtered (300–6,000 Hz; Krohn-Hite). The signals were monitored in real time with an oscilloscope (DL1540; Yokogawa) and via speakers connected to an audio amplifier and were sampled at 50 ks/s to computer disk (Power1401 and Spike2 software; CED).

Before conducting the recordings reported below, all animals underwent electrophysiological mapping sessions to identify the location of the GPe within the recording chambers. The extent of GPe along the electrode tracks was identified by the depth of the structure within the chamber, and by using the characteristic sequence of electrophysiological recordings along the electrode track, starting with low-frequency striatal cells at dorsal recording locations, to “border cells” between the striatum and GPe, and GPe with a large proportion of cells displaying high-frequency firing. The lower border of GPe was identified by using the electrode penetration depth, as well as by encountering the transition into the internal pallidal segment (GPi, characterized by sustained irregular high-frequency firing). When the transition into GPi was not apparent, only cells within 2 mm from the ventral border of the putamen were considered to be in GPe. Well-isolated single GPe units were then recorded for 5–60 min.

In cases in which ECoG signals were available, these were amplified (2,500) and filtered (0.3–1,000 Hz) using a custom-built amplifier/filter unit. The signals were sampled at 5 ks/s (Power 1401 and Spike2 software).

### Induction and assessment of parkinsonism

Five of the seven animals were rendered moderately parkinsonian by weekly injections of MPTP (0.2–0.8 mg/kg, i.m., Sigma-Aldrich). A description of the MPTP administration procedure is provided in doi.org/10.17504/protocols.io.ewov1o1polr2/v1. Table 1 indicates the cumulative dose of MPTP administered per animal. The severity of parkinsonism was assessed weekly, using rating scales for parkinsonism and computer-assisted behavioral scoring methods as described in detail in our previous studies (Galvan et al., 2010; Galvan et al., 2014) and in doi.org/10.17504/protocols.io.x54v928ppl3e/v1. Recordings in the parkinsonian state started at least 6 weeks after the last injection of MPTP, once the animals were stably parkinsonian.

### Perfusion and tissue processing

After completion of the recording experiments, the monkeys were deeply anesthetized with an overdose of pentobarbital (100 mg/kg, i.v.) and then transcardially perfused with oxygenated Ringer's solution followed by fixative solution consisting of 4% paraformaldehyde and 0.1% glutaraldehyde (doi.org/10.17504/protocols.io.5jyl8jn67g2w/v1). The brains were subsequently extracted from the skull and placed in 4% paraformaldehyde for 24 h. Slabs of brain tissue were then cut in 60-µm-thick coronal sections using a vibrating microtome.

A series of sections containing GPe tissue was stained for Nissl substance, or the neuronal marker microtubule-associated protein 2 (MAP2), to visualize tissue defects resulting from the electrode penetrations. In MPTP-treated monkeys, another series of sections containing the striatum and the substantia nigra were stained for tyrosine hydroxylase (TH) to evaluate the degree of nigrostriatal dopaminergic denervation (compared with untreated controls). As previously described (Galvan et al., 2010, 2011; Galvan et al., 2014), the sections were incubated with anti-TH antibodies (Millipore MAB318 or Millipore AB152, 1:1,000 and 1:2,000 dilutions, respectively), whose locations were subsequently revealed using biotinylated secondary antibodies and the avidin-biotin complex (ABC) method.

The sections were then mounted, coverslipped, and scanned (at a maximal magnification of 20×) with an Aperio Scanscope CS system (Leica). For quantification of the TH staining, images of six sections containing the striatum were assessed in each animal. In each section, 1–3 ROIs of the caudate (dependent on caudate size) and 4 ROIs of the putamen were included in the analysis. The images were inverted, and the mean density was calculated using ImageJ (Schneider et al., 2012). The values were compared against those obtained from four healthy control animals (tissue obtained from our rhesus macaque brain bank).

### Analysis of electrophysiological data

#### Sorting of spikes and quality control

Spike sorting was performed offline with a waveform matching algorithm, followed by principal component analysis (Spike2). Interspike interval (ISI) distribution histograms were constructed to verify the recording and sorting quality for each identified unit. Records in which more than 2% of all ISIs were shorter than 2 ms were excluded from further analysis. For all subsequent analyses, the ISI data were exported to Matlab (MathWorks). As an additional quality measure, we first calculated “isolation scores” for each minute of recording (Joshua et al., 2007). The isolation scores range from 0 to 1. To ensure stable isolation quality throughout the recording, we included only cases in which an isolation score of ≥0.85 was maintained for each minute of the recording.

To analyze variations in the waveform of individual units during the recording, each recorded action potential waveform of a given neuron was compared with the mean waveform of the same neuron within a reference range, defined as the central 500 recorded action potential waveforms of the neuron. We calculated the mean and standard deviation of the sum of absolute differences from this average for all waveforms within the reference range. Next, the same sum of absolute difference was calculated for all available spike waveforms. We then calculated the proportion of waveforms for which the sum of absolute differences was outside of the mean ± 2.5 SD range of those of the reference range. Cells for which this proportion was <10% were deemed acceptable for further analysis.

#### Descriptors of neuronal firing patterns

**Figure 1.**
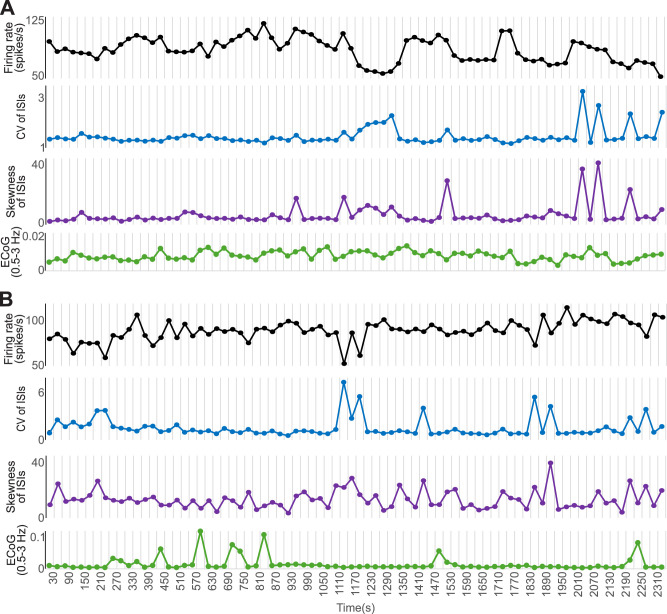
Temporal variability in firing rate and pattern of GPe neurons. ***A***, Firing rate (black), CV of ISIs (blue), and skewness of the ISI distribution (purple) from a GPe neuron, presented in non-overlapping 30 s bins. The power of the 0.5–3 Hz spectral range in simultaneously collected ECoG is shown in green. ***B***, Same as ***A***, for a second neuron collected in the same animal, on a different recording day. The time labels on the bottom trace of panel ***B*** apply also to ***A***.

**Figure 2.**
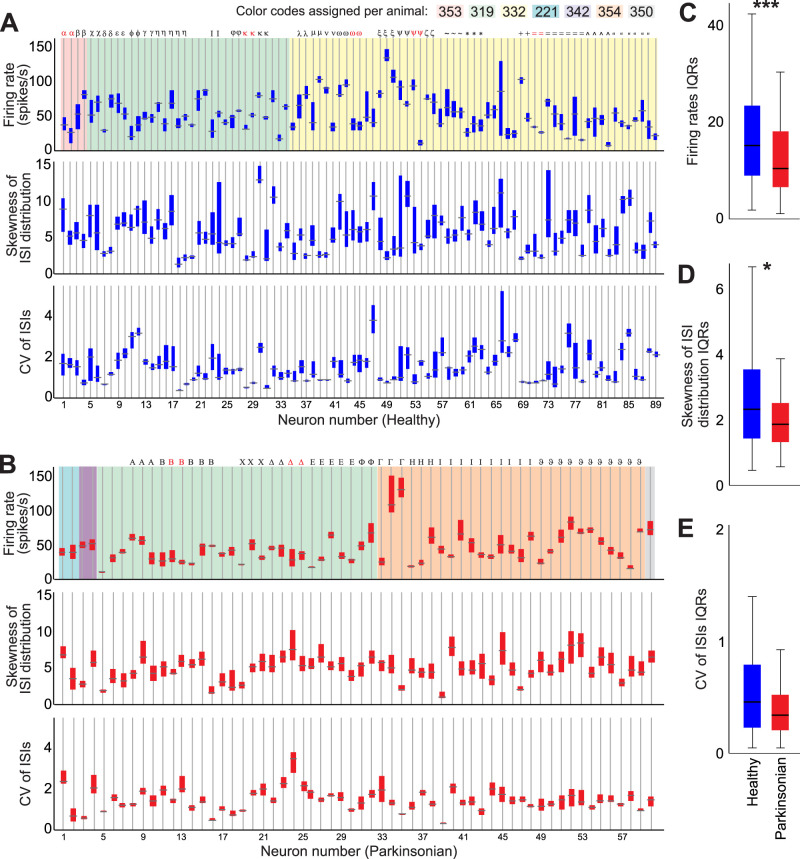
Range of firing rates and measures of the regularity of firing in recordings of GPe neurons in the healthy and parkinsonian states. ***A***, ***B***, Ranges of firing rates, skewness of the ISI distribution, and CV of ISIs in single GPe cells that were recorded in the healthy (blue bars, ***A***) and parkinsonian (red bars, ***B***) states. Each bar represents one neuron. In each case, the horizontal line indicates the median, and the top and bottom indicate the 25th and 75th percentile, respectively. The color shading indicates neurons recorded in the same animal. The symbols on top of each bar indicate cells recorded in the same recording track. Neurons recorded at the same site are marked in red. An absence of symbols indicates recording sessions with only one cell. To allow comparisons, the first 7,000 ISIs of each recording were used, and the data were binned into consecutive epochs of 500 ISIs each. In ***C–E*** the IQRs of firing rates, distribution of ISI skewness, or CV of ISIs were compared among groups (**p < 0.01, *p < 0.05, Mann–Whitney test, 89 healthy and 60 parkinsonian cells). In the box plots, the horizontal line represents the median across cells, and the top and bottom of the box represent the 25th and 75th percentile ranges. Whiskers extend to the 5th and 95th percentiles.

While some of the analyses were conducted using the full length of the recordings, for others we selected the maximal number of ISIs that were common to all files (i.e., 7,000 ISIs), counted from the start of the respective record. To study the variability of firing pattern parameters over time, data was either binned in 30 s data segments (Figs. 1, 5, 6) or in segments spanning 500 ISIs (Figs. 2, 4). In addition to several standard features such as the mean or the CV of ISIs, we also calculated the skewness of the ISI distribution, a nonparametric measure of the asymmetry of this distribution that reflects the variability of firing (Shinomoto et al., 1999; Katabi et al., 2022).

**Table 2.** Firing parameters of GPe cells

	Healthy (*n* = 89)	Parkinsonian (*n* = 60)	Mann–Whitney *U*	*p* value (2-tailed)	Effect size (*r*)
Duration of recording (s) to reach 7,000 ISIs	155.8 (107.8–214.4)	178 (133.2–249.6)	2,224	0.084	0.14
Firing rate (spikes/s)	44.9 (32.6–65)	39.3 (28–52.6)	2,224	0.084	0.14
CV of ISIs	1.9 (1.1–2.6)	1.6 (1.3–2.1)	2,268	0.12	0.13
Skewness of ISI distribution	9.5 (6.1–13.3)	7.4 (5.4–9)	1,968	0.007	0.22
ISI IQR	12.3 (9–18.2)	14.6 (10.9–23.8)	2,106	0.029	0.18
iPS, 1–3 Hz	0.04 (0.03–0.06)	0.05 (0.02–0.06)	2,521	0.564	0.05
iPS 3–8 Hz	0.05 (0.04–0.06)	0.06 (0.04–0.07)	2,206	0.073	0.15
iPS 8–13 Hz	0.04 (0.03–0.04)	0.04 (0.03–0.05)	2,166	0.051	0.16
iPS 13–30 Hz	0.11 (0.09–0.13)	0.12 (0.1–0.13)	2,427	0.347	0.08
iPS 30–100 Hz	0.75 (0.71–0.79)	0.72 (0.68–0.79)	2,150	0.044	0.16
Burst index	0.025 (0.006–0.073)	0.041 (0.017–0.073)	2,314	0.169	0.11
Pause index	0.09 (0.02–0.18)	0.07 (0.02–0.11)	2,246	0.1	0.13

The table describes the firing parameters of GPe cells in the healthy and parkinsonian conditions. The analysis is based on 7,000 ISIs each. Shown are medians followed by IQRs, in parenthesis. Abbreviations: CV, coefficient of variation; ISI, interspike interval; iPS, integrated power spectrum, IQR, interquartile range.

Several measures of phasic firing characteristics were also generated. To detect bursts of neuronal activity, we used the “surprise” method (Legendy and Salcman, 1985), with a “surprise” value of 10, as recently used in an analysis of similar data (Katabi et al., 2022). The “burst index” was the proportion of the cell’s recording time occupied by bursts. Pauses in firing were defined following the algorithm described by Katabi et al. (2022). This method is a variant of the “surprise” method, in this case assessing the improbability of finding less than a certain number of spikes in a data segment with a given firing rate. The algorithm first identifies long ISIs (the “minimal core interval”) and then maximizes the surprise value by adding up to 5 ISIs (in either direction). Overlapping and immediately adjacent pauses are combined. The eventually defined pauses need to exceed a predefined length (the “minimal length of the final pause”). The initially assigned values for the minimal core interval was 250 ms and that of the minimal length of the final pause 300 ms. Both values were adjusted for each analyzed data segment, to account for the fact that pauses would be expected to be longer in slow firing cells than in fast firing cells. We multiplied the minimal core interval value and the minimal length of pauses with the ratio of the average ISI length and an arbitrarily chosen “baseline ISI” (set to 20 ms). In this study, we report the proportion of total recording time occupied by pauses (as defined by the algorithm).

Power spectral analyses were done with NeuroSpec 2.0 functions (Halliday et al., 1995; Nielsen et al., 2005). For each neuron, the raw spectra were integrated in the 1–3, 3–8, 8–13, 13–30, and 30–100 Hz ranges and the resultant values expressed as a fraction of the power in the entire 1–100 Hz band.

#### ECoG analysis

For nine cells, simultaneous neuronal and ECoG recordings were available, allowing us to examine whether GPe neuron firing patterns and their classification changed with the state of wakefulness. In these cases, we evaluated the correlation between the firing rate, the ISI skewness, and the power of the slow waves. Spectrograms of the ECoG signals were calculated for 30 s windows using the multitaper time–frequency method [implemented in the Chronux open source toolbox for Matlab (https://chronux.org/), with a moving window width of 1 s and a window shift of 0.25 s]. The frequency resolution of the spectra was 0.0191 Hz/bin. We extracted the sum of the power spectral density in the 0.515–2.9945 Hz range (as a proxy for drowsiness) and then evaluated the Spearman correlation between these values and corresponding measurements of firing rates and ISI skewness. These recordings were 2,340–3,630 s long. Thus, binning in 30 s windows provided 78–121 points for correlation testing.

#### Determination of the precision of firing rate estimates

We used our recordings of GPe neuron firing to analyze the variability of estimates of the overall firing rate (i.e., the number of spikes in the entire record, divided by the length of the record), if such estimates were based on analyses of shorter segments. To examine the variability of estimates based on a specific segment duration, 15 randomly chosen, non-overlapping data segments of the desired shorter duration were extracted from the overall data. For each cell, this analysis was performed using segments of ascending duration, in steps of 250 ms, up to the largest duration still allowing the use of 15 non-overlapping segments. These data were then used to estimate the minimum and maximum value that would result from recording the same cell for the chosen duration at different times. From these, the maximal positive or negative deviation from the overall firing rate was determined as a measure of the accuracy of the firing rate estimate. This serial analysis allowed us to determine the length of data segments (or spike counts) needed to arrive at consistent firing rate estimates within 5 or 10% of a value that represents the neuron’s overall firing rate (5 and 10% were arbitrarily chosen). If the error estimates did not steadily decline with longer segment lengths, we chose the segment length beyond which all longer segments would yield estimates that were within 5 or 10% of the overall value, respectively.

#### “Path length” analysis and determination of functional states

These analyses were done to examine the stability of the neurons' functional state. Each cell's record was divided into consecutive 30 s intervals, overlapping by 10 s, and firing rates, burst indices, and pause indices were determined for each segment. Envisioning these parameters in a 3D space (Fig. 5*A*,*B*), plotted against axes of equal length representing a firing rate range of 0–120 spikes/s, a burst index range of 0–0.2, and a pause index range of 0–0.8, the cell can be seen as traveling along a trajectory from data segment to data segment. As a measure of state variability, we calculated the average path length and its SD.

We also studied whether segments fulfilled criteria for “HFD-P” or “LFD-B” firing, and whether such designations remained stable during the cell’s record. Studying all available 30 s segments from all cells, we first determined cutoff criteria for “high frequency” and “low frequency” firing, as spike rates at or above the 50th percentile of rates (50 spikes/s), or below the 25th percentile of rates (33 spikes/s), respectively. Threshold pause indices or burst indices were used to define whether a cell was “bursting” or “pausing.” These thresholds were set as the median of pause or burst indices across all available segments, resulting in a pause index cutoff value of 0.03 and a burst index cutoff value of 0.0085. For a recording segment to be considered to represent the “LFD-B” state, it had to fulfill the criteria for a low firing rate and for “bursting.” Likewise, to qualify as an “HFD-P” segment, the firing rate had to be above the high-frequency range, and the pause index had to be above its threshold. Segments of recording that did not fulfill these criteria were considered “undetermined.”

#### Statistics

Summary data are reported as medians with interquartile ranges (IQRs). We used IBM SPSS Statistics to build graphs and to conduct statistical analyses. Mann–Whitney and Wilcoxon tests were used for nonpaired and paired comparisons, respectively. Statistical significance was defined at *p* < 0.05. Effect sizes (*r*) were calculated by dividing the standardized test statistic (*Z* value) from the Mann–Whitney test by the square root of the total number of cases (Jesussek and Volk-Jesussek, 2025). The values of the Mann–Whitney tests, with the corresponding *p* and *r* values (when *p* < 0.05) are provided in the Results section. The number of cases used for each analysis is provided in the legend to each figure.

## Results

### Induction of parkinsonism

Animals that received MPTP developed moderately severe parkinsonism, with parkinsonism rating scores of 15 [13.5-15.5] (median and [IQR], maximal value is 30). Postmortem immunohistochemistry showed that the density of the remaining TH-positive fibers in the post-commissural lateral putamen (the region most affected by the dopaminergic denervation induced by MPTP; Masilamoni and Smith, 2018) was 17% [12–22], compared with control animals.

### Database

The analysis was based on 89 GPe cells recorded in the healthy state and 60 GPe cells recorded in the parkinsonian state. One animal was recorded in both states (contributing 30 and 28 neurons in the healthy and parkinsonian states, respectively). The median duration of the recordings was 899 s [618–1,219] (1,202 s [805–1,507] for healthy animals; 655 s [545–721] for parkinsonian animals). The median number of ISIs collected for each cell was 38,226 [24,208–71,053] (58,325 [33,240–114,672] for healthy animals; 30,076 [13,875-37,379] for parkinsonian animals).

All neuronal recordings included in the analysis were deemed stable based on two criteria: (1) the unit isolation remained constant (based on isolation score > 0.85, calculated minute-by-minute) and (2) each spike's waveform changes remained within mean ± 2.5 SDs of the reference range for at least 90% of all spikes (see Materials and Methods). To compare features of firing between GPe cells that were recorded in the healthy and parkinsonian states, we used the first 7,000 ISIs of each recording (Table 2). GPe neurons recorded in the parkinsonian state showed significant changes in the variability of firing compared with the healthy state, as revealed by a decreased skewness of the ISI distribution, an increase in the ISI interquartile range, and a reduction in the integrated power spectrum in the 30–100 Hz band. Other parameters were not statistically different between the two stages.

### Variability of firing in individual GPe cells

The firing rates and patterns of spontaneously active GPe cells varied considerably over time. As examples, we show data from two recordings obtained in the same healthy animal on different days (Fig. 1). The data were binned in non-overlapping segments of 30 s each. During the recorded time, the firing rate of both neurons varied with time. Note that we calculated two descriptors of the regularity of firing. One of them, the CV of ISIs, is a commonly used measure to study the regularity of firing. Given the non-normality of ISI distributions, we used a second descriptor, i.e., the skewness of the ISI distribution. The skewness provides an additional characterization of regularity and has been incorporated in recent studies of neuronal firing (Katabi et al., 2022). The firing of the neuron in panel A was relatively stable in both measurements, while the cell shown in B had marked variability in firing regularity, most noticeable in the skewness of the ISI distribution. In a fraction of our recordings, ECoGs were collected simultaneously with the single-unit data. We used the power in the 0.5–3 Hz band as a proxy for the state of wakefulness (Fig. 1, green traces) and investigated if the ECoG slow wave activity was correlated with firing rate or with measurements of firing regularity (Table 3). Except for one case in which the ECoG activity was weakly correlated with the skewness of the ISI distribution, we did not find significant correlations, indicating that the changes in firing rates or the regularity of firing were not a consequence of (subtle) changes in the state of alertness of the animals.

**Table 3.** Correlations between parameters of firing and ECoG data

Cell ID	Firing rate vs low-frequency ECOG power	Skewness of ISI distribution vs low-frequency ECOG power	CV of ISIs vs low-frequency ECOG power
353_004a	0.03 (*p* = 0.77)	0.09 (*p* = 0.35)	−0.06 (*p* = 0.48)
353_004b	−0.05 (*p* = 0.6)	0.02 (*p* = 0.84)	0.15 (*p* = 0.09)
353_007	−0.16 (*p* = 0.15)	0.02 (*p* = 0.88)	0.08 (*p* = 0.48)
319_014	−0.05 (*p* = 0.68)	0 (*p* = 0.99)	0.07 (*p* = 0.52)
319_031	0.12 (*p* = 0.31)	0.24 (*p* = 0.03)	0.09 (*p* = 0.43)
319_065	−0.03 (*p* = 0.81)	−0.1 (*p* = 0.4)	−0.08 (*p* = 0.48)
332_005	0.09 (*p* = 0.3)	0.13 (*p* = 0.16)	0.01 (*p* = 0.89)
332_010	0.14 (*p* = 0.22)	0.03 (*p* = 0.79)	−0.1 (*p* = 0.41)
332_015	0.06 (*p* = 0.61)	−0.06 (*p* = 0.58)	−0.23 (*p* = 0.05)

Correlations between firing rates, ISI distribution skewness, or the CV of the ISIs, and ECoG 0.5–3 Hz spectral power. Values shown are Spearman's rank correlation coefficients and *p* values.

Across animals, days, and health states, individual GPe neurons had a wide range of firing rates, CV of ISIs, and distributions of ISI skewness (Fig. 2*A*,*B*). In Figure 2, color shadings refer to recordings from individual monkeys. The recordings are presented in chronological order with earlier recordings to the left. The symbols above each bar indicate recordings conducted on the same day. No consistent relation was observed in the variability in firing parameters observed in neurons collected across recording days in the same animal.

The ranges of firing rates of individual GPe neurons (expressed as IQRs) were significantly larger in the healthy (median 15.3, IQR [9–23.5]) than in the parkinsonian state (10.5 [6.7–18.1], Mann–Whitney *U* = 1,954, *p* = 0.006, *r* = 0.22; Fig. 2*C*). Similarly, the firing regularity, as measured by the skewness of ISI distributions, occupied a wider range in the healthy (2.3 [1.4–3.5]) than in the parkinsonian condition (1.8 [1.3–2.5], Mann–Whitney *U* = 2,051, *p* = 0.017, *r* = 0.19; Fig. 2*E*).

We calculated the correlation between the firing rate and the skewness of ISI distribution or the CV of ISIs. The firing rate was consistently negatively correlated with the CV, in both the healthy and parkinsonian states (Spearman *r* = −0.37 and −0.2, healthy and parkinsonian respectively, both *p* < 0.001). However, it was not correlated with the skewness of ISI distribution in the healthy state and only weakly correlated in the parkinsonian state [Spearman *r* = 0.03 (*p* = 0.3) and 0.08 (*p* = 0.02), healthy and parkinsonian, respectively]. Thus, the skewness of the distribution of ISIs provides a measure of firing regularity that is less dependent on the firing rate. We chose this measurement for the remaining analyses.

### Precision of firing rate estimates

It is intuitively obvious that short recordings provide less precise estimates of firing rates of the recorded cells than long recordings, but the extent to which this matters is generally not known for specific recording situations. Taking advantage of the long duration of recordings in our study, we calculated the shortest segment length of recordings that would provide a firing rate estimate within 5 or 10% of the firing rate estimate based on the entire available data.

**Figure 3.**
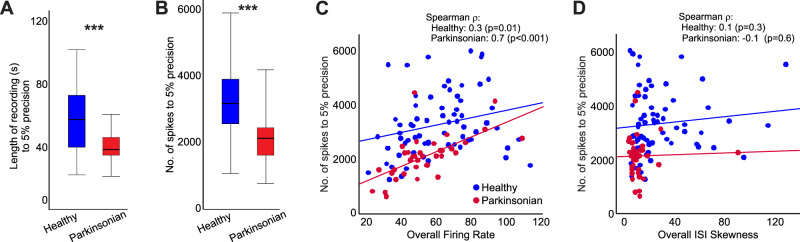
Determinants of the accuracy of firing rate estimates of GPe cells. ***A***, ***B***, The number of spikes (***A***) and the length of recordings (***B***) needed to reach an estimate within 5% of the overall firing rate for each neuron. The data are compared between healthy and parkinsonian animals. (***p < 0.001, Mann–Whitney test). ***C***, ***D***, Correlation between the number of spikes needed to reach an estimate within 5% of precision and the overall firing rate (***C***) or skewness of the ISI distribution (***D***). The analysis is based on data from 69 cells recorded in the healthy state, and 36 cells recorded in the parkinsonian state. Box plots conventions same as in Figure 2.

In healthy animals, we found that a recording with a median duration of 57 s [39–72] (or 3,190 spikes [2,590–3,941]) was the minimum required to generate a firing rate estimate that deviated <5% from the measured overall firing rate, determined by dividing the number of spikes by the total recording time (Fig. 3*A*,*B*). A recording with a median duration of 15 s [11–24] or 795 spikes [646–930] would allow estimation within 10% precision of the overall firing rate (data not shown).

In the parkinsonian state, the minimal recording time to reach a 5% precision was 38 s [34–45], significantly lower than in the healthy state (Mann–Whitney *U* = 622, *p* < 0.001, *r* = 0.41). Similarly, the number of spikes to reach a 5% precision was smaller in parkinsonian than in healthy monkeys (2,157 [1,651–2,468] spikes, Mann–Whitney *U* = 458, *p* < 0.001, *r* = 0.51; Fig. 3*A*,*B*). The recording times and numbers of spikes needed to reach a 10% precision of firing rate estimates did not differ between the healthy and parkinsonian states (18.75 s [13–24], Mann–Whitney *U* = 2,150, *p* = 0.088, compared with the healthy condition; number of spikes 728 spikes [617–841], Mann–Whitney *U* = 2,208, *p* = 0.139, compared with the healthy condition).

We found a significant correlation between the overall firing rate and the number of spikes needed to predict firing rates at 5% precision [Spearman *r* = 0.3 (*p* = 0.01) and 0.7 (*p* < 0.001) for healthy and parkinsonian, respectively; Fig. 3*C*]. However, there was no correlation between the overall skewness of the ISI distribution and the number of spikes to 5% precision [Spearman *r* = 0.1 (*p* = 0.3) and −0.1 (*p* = 0. 6) for the healthy and parkinsonian states, respectively; Fig. 3*D*]. The duration of recording needed to achieve a 5% precision was negatively correlated to the overall firing rate in both healthy [Spearman *r* = −0.5 (*p* < 0.001)] and parkinsonian [(Spearman *r* = −0.3 (*p* = 0.03)] conditions (data not shown).

### GPe neurons display a wide spectrum of firing rates and variability

**Figure 4.**
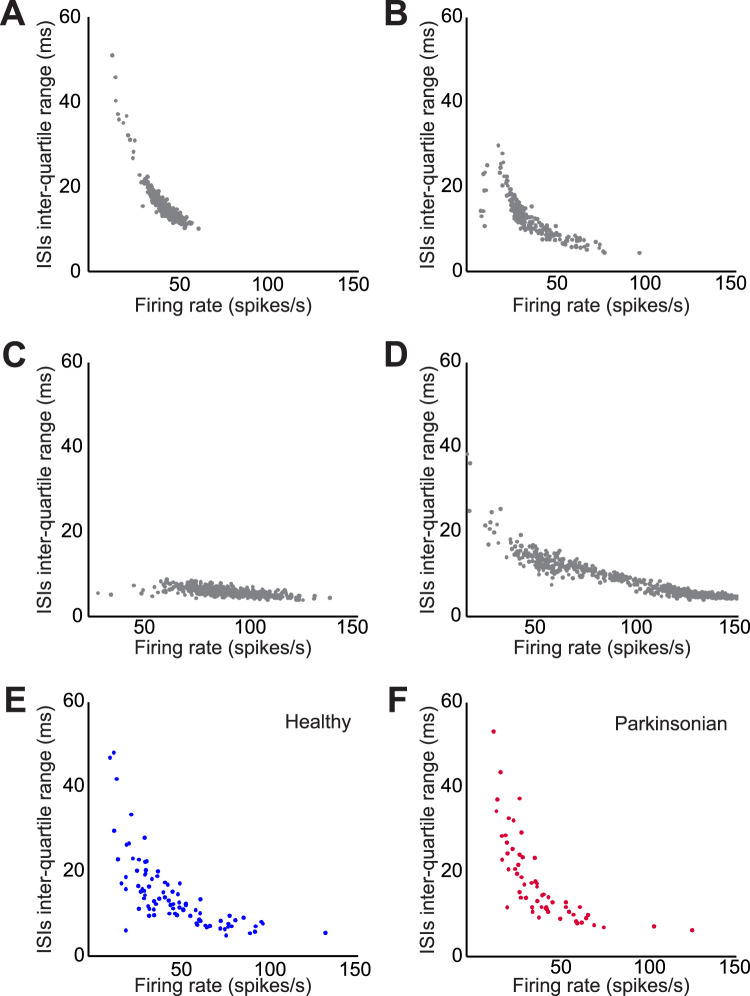
GPe cells cover a wide spectrum of firing rates and variabilities. ***A–D***, IQRs of ISIs from four example cells are plotted against the corresponding firing rates. Each symbol represents analyses of non-overlapping segments of 500 ISIs from the same cells. Each cell was recorded for at least 3,200 s. ***E***, ***F***, Summary data from all neurons in the healthy and parkinsonian states (in this case, each symbol is based on the first 7,000 ISIs of individual cells).

In vivo and in vitro recordings in mice have demonstrated that the firing rate and regularity of GP neurons are highly heterogeneous. Applied to a population of GPe recordings, these analyses found that the firing rate and variability of firing had a consistent (hyperbolic) relationship (Deister et al., 2013; Abdi et al., 2015). We conducted a similar analysis using our database of GPe cells recorded in monkeys. We generated scatterplots of the IQR of the ISIs against the event rate of short segments of the data. In some cases, the firing rate and regularity of individual cells covered only a portion of the spectrum of firing rates and variability estimates generated by GPe cells in general (Fig. 4, compare *A–C*, *E*,*F*), while other neurons traversed a broader spectrum of firing rates and variabilities (Fig. 4*D*), similar to what was described in the aforementioned rodent studies (Deister et al., 2013). As a population, GPe cells covered a wide spectrum of firing rates and variability, both in the healthy and parkinsonian conditions (Fig. 4*E*,*F*).

### Individual GPe neurons display fluctuations in the proportion of bursts and pauses in firing

Given the traditional classification of GPe neurons into cells showing either high-frequency firing interspersed with pauses (HFD-P), or cells showing low-frequency discharge with bursts (LFD-B; DeLong, 1971; Katabi et al., 2022), we studied whether GPe neurons remained stable in an HFD-P or LFD-B state or whether they could move between these states.

**Figure 5.**
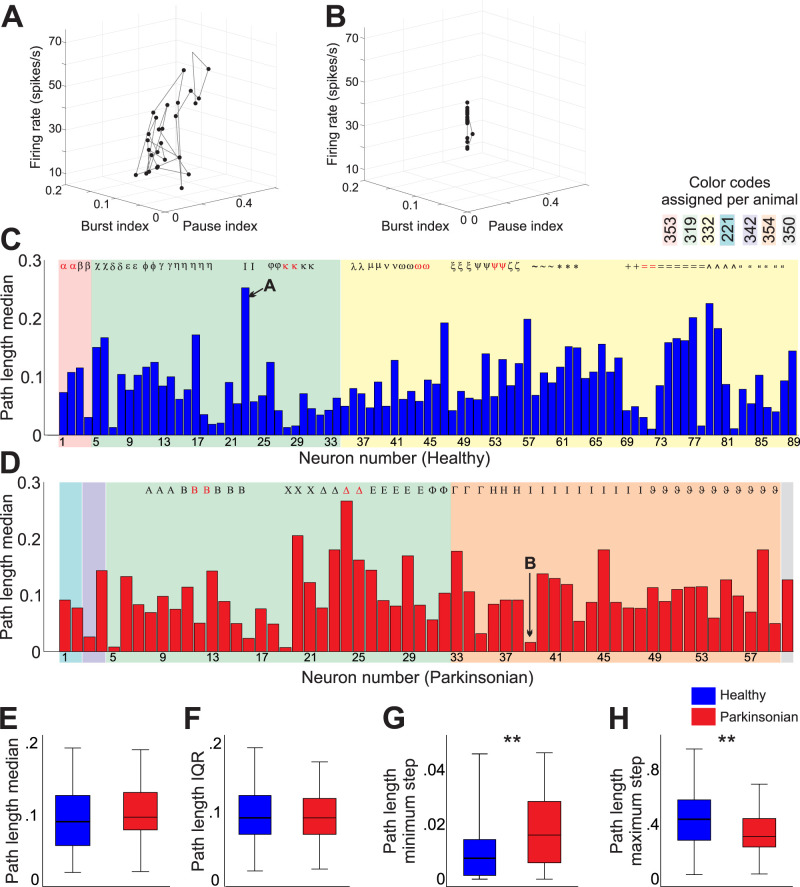
GPe neurons occupy a continuum of firing rates, bursts and pauses. ***A***, ***B***, 3D plots of the relationship between firing rates, burst index, and pause index for two example GPe cells (recorded for 599 and 679 s, respectively). Each symbol represents a bin of 30 s of firing, with a 10 s overlap. The order of cells, color shades, and symbols are the same as in Figure 2. ***C***, ***D***, The IQR of the steps taken by each cell in their individual paths in the 3D space for neurons recorded in the healthy (***C***) or parkinsonian (***D***) states. Cells presented in ***A*** and ***B*** are noted by arrows. The total time recorded for each cell was used for the analysis. ***E–H***, Comparisons of the median step in the path, the IQRs of all steps, and the minimum and maximum steps between healthy and parkinsonian cells (**p < 0.01, Mann–Whitney test, 89 healthy and 60 parkinsonian cells). Box plot conventions same as in Figure 2.

We first calculated how much each individual GPe neuron “traveled” in a 3D space constructed by the firing rate, the burst index (proportion of time in bursts) and the pause index (proportion of time spent in pauses), calling this parameter the “path length” (Fig. 5). We found that many neurons traveled a long path in this 3D space, while others remained more constrained (compare the two example neurons shown in Fig. 5*A*,*B*, each recorded for ∼10 min).

We plotted the median of the steps taken between each 30 s data point for each cell (Fig. 5*C*,*D*). Larger values indicate longer paths, as in the neuron shown in Figure 5*A*, smaller values noted shorter paths, i.e., neurons with less variation across firing rates, burst, and pause prevalence, as in the example shown in Figure 5*B*. Variable median path lengths were found across monkeys and recordings in the same monkey.

There were no significant differences in the median (0.08 [0.05–0.12] and 0.09 [0.07–0.13] for healthy and parkinsonian conditions, respectively, Mann–Whitney *U* = 2,340, *p* = 0.2) or the IQRs of the steps taken by cells recorded in the healthy or parkinsonian states (0.09 [0.07–0.12] for both healthy and parkinsonian conditions, Mann–Whitney *U* = 2,569, *p* = 0.7; Fig. 5*E*,*F*). However, compared with neurons recorded in the parkinsonian state, GPe neurons in healthy monkeys displayed smaller minimal steps (healthy, 0.01 [0, 0.01]; parkinsonian 0.02 [0.01, 0.03], Mann–Whitney *U* = 1,833, *p* = 0.001, *r* = 0.25) and larger maximal steps (healthy, 0.44 [0.29–0.58]; parkinsonian 0.31 [0.23–0.44], Mann–Whitney *U* = 1,869, *p* = 0.002, *r* = 0.24; Fig. 5*G*,*H*).

However, exhibiting a long path in the 3D space does not necessarily indicate fluctuations between HFD-P and LFD-B states. Therefore, we investigated the possibility that GPe neurons transition across these functional states, using the criteria for these states that were mentioned in the Materials and Methods. A GPe neuron was defined as entirely HFD-P or LFD-B if it spent at least 75% of its time segments in the corresponding state with <10% of time in the other. Using this (arbitrary) criterion, among the 149 neurons in our database, only 10 were classified as HFD-P and 12 as LFD-B. A previous publication described that GPe cells categorized as HFD-P have shorter duration action potential waveforms than LFD-B (Katabi et al., 2022). In our results, the median action potential duration of the 10 cells identified as fully HFD-P was 0.2 ms [0.17–0.22], not significantly different from the values measured in LFD-B cells (0.2 ms [0.18–0.23], Mann–Whitney *U* = 55.5, *p* = 0.7).

**Figure 6.**
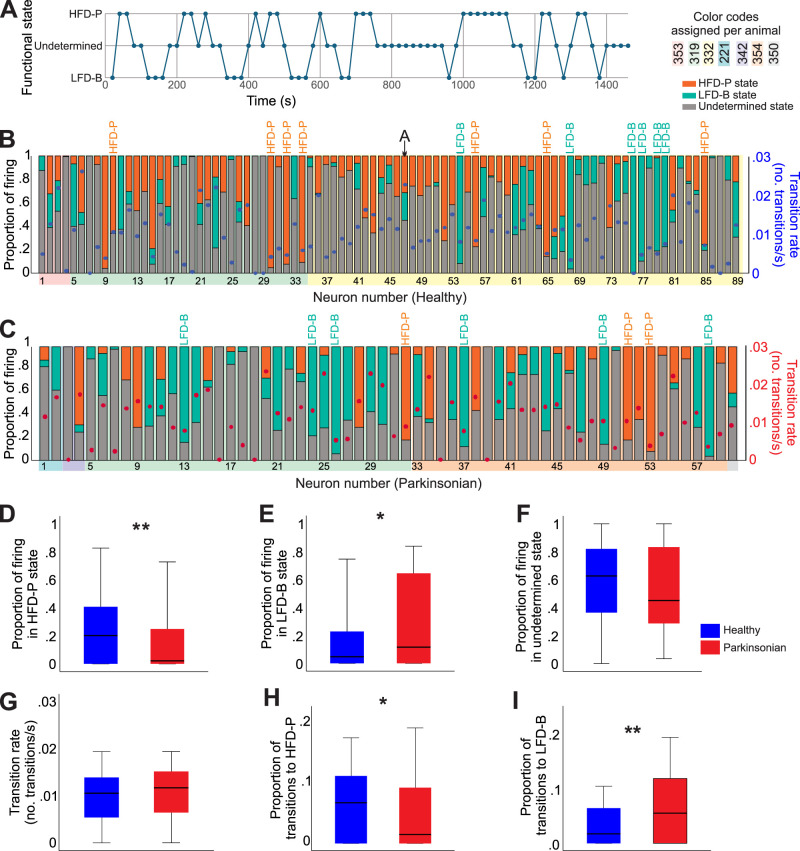
Individual GPe cells transitions between HFD-P and LFD-B states. ***A***, Graphical representation of an example neuron which firing transitioned between HFD-P, undetermined, and LFD-B states during the time recorded. Each data point represents a bin of 30 s of firing, with a 10 s overlap between segments. ***B***, Proportion of time that individual neurons recorded in the healthy state spent in each of the three functional states, each bar represents a neuron. The order of cells and the color shades are the same as in Figure 2. The transition rate (number of transitions between states/s) is represented by a dot in each column and the axis on the right. ***C***, same as ***B*** for GPe cells recorded in the parkinsonian condition. ***D–I***, Comparisons of the proportion of firing spent in the HFD-P, the LFD-B, and the undetermined states, the transition rate, proportion of all transitions to HFD-P and to LFD-B among groups (*p < 0.05, **p < 0.01, Mann–Whitney test, 89 cells recorded in the healthy state and 60 in the parkinsonian state). Box plot conventions same as in Figure 2.

Thus, most cells spent only a fraction of the recorded time in the defined HFD-P or the LFD-B states (see example in Fig. 6*A*). Instead, during most of the recording time, the majority of GPe neurons were found in an undetermined state, i.e., not fulfilling the two criteria needed to be characterized as HFD-P or LFD-B (Fig. 6*B*,*C*). GPe neurons recorded in the parkinsonian condition spent much less time in the HFD-P state (healthy, 0.2 [0, 0.4]; parkinsonian, 0.02 [0,0.25], Mann–Whitney *U* = 1,949, *p* = 0.004, *r* = 0.22) and more time in the LFD-B state (healthy, 0.05 [0, 0.2]; parkinsonian, 0.12 [0, 0.64], Mann–Whitney *U* = 2,143, *p* = 0.037, *r* = 0.16; Fig. 6*D*,*E*). The proportion of time spent in the undetermined state was not different among groups (healthy, 0.6 [0.3–0.8]; parkinsonian, 0.4 [0.3–0.8], Mann–Whitney *U* = 2476, *p* = 0.4; Fig. 6*F*).

GPe neurons transitioned across stages at a rate of 0.01 [0.005–0.013] and 0.011 [0.006–0.015] transitions/s, for recordings in the healthy and parkinsonian state, respectively (Fig. 6*G*; not significantly different, Mann–Whitney *U* = 2,420, *p* = 0.3). We also calculated the number of any type of transition to HFD-P or LFD-B and expressed this number as proportion to account for the different recording lengths. In agreement with the higher proportion of HFD-P states in healthy animals, the proportion of transitions to this state was higher, compared with parkinsonian monkeys (healthy, 0.07 [0, 0.1]; parkinsonian, 0.01 [0, 0.1], Mann–Whitney *U* = 2,114, *p* = 0.03, *r* = 0.23; Fig. 6*H*). As expected, the proportion of transitions to the LFD-B was higher in GPe neurons from parkinsonian monkeys (healthy, 0.02 [0, 0.06]; parkinsonian, 0.05[0, 0.12], Mann–Whitney *U* = 2,008, *p* = 0.008, *r* = 0.27; Fig. 6*I*).

Regardless of condition, GPe cells very rarely transitioned directly from HFD-P to LFD-B or vice versa: we found these transitions in only 10 healthy and 6 parkinsonian cells, and on average they accounted for <3% of all transitions. When we considered indirect transitions (i.e., transitions from HFD-P to LFD-P or vice versa through a single undetermined epoch), we found they accounted for <4% of total transitions, in either condition. Twenty healthy and 11 parkinsonian cells showed this type of transition.

## Discussion

Using long recordings of GPe neurons in healthy and parkinsonian monkeys, we show that the heterogeneity of firing patterns in this nucleus is unlikely to reflect categorical differences between neurons but that it may reflect temporal variations in the firing rates and patterns of individual cells. Given these temporal fluctuations, long recordings are necessary to accurately represent the range of firing characteristics of GPe neurons.

While substantial variability of firing rates and patterns was identified in most GPe neurons (Fig. 2), the extent of these fluctuations differed between neurons. As a population, however, GPe cells cover a large spectrum of firing rates and patterns. Our results agree with previous in vitro and in vivo recordings of pallidal neurons in rats (Bugaysen et al., 2010; Deister et al., 2013).

Temporal variation in GPe firing may reflect both intrinsic and extrinsic factors, such as ion channel distribution, ionic current fluctuations, and synaptic connectivity (Kita et al., 2004; Kita et al., 2005; Gunay et al., 2008; Deister et al., 2013; Wilson, 2013). Additionally, it is known that GPe consists of different genetic subtypes which may differ in their electrophysiological properties (Courtney et al., 2023).

The variability of GPe firing may serve functional roles. For example, it may contribute to oscillatory activity or determine the dynamic range of information transmission, for example, to encode movement parameters (DeLong et al., 1984; Hamada et al., 1990; Yoshida and Hikosaka, 2025).

Given that some GPe neurons vary their firing in seconds or minutes, relatively long-duration recordings may be needed to accurately capture the behavior of these cells. We found that, in healthy monkeys, recordings with a median duration of ∼60 s (or ∼3,200 spikes) are required to estimate a cell's firing rate with <5% deviation from the overall firing rate, as defined in the Methods. Shorter recordings may add variability to comparisons of firing rates across different behavioral conditions (e.g., comparisons between healthy and parkinsonian states). In parkinsonian monkeys, shorter recordings (∼40 s) were needed to achieve estimates with similar precision, probably due to the smaller range of variability in this condition. These considerations may help interpret past GPe recording studies and guide future work, particularly when long recordings are impractical (e.g., intraoperative human recordings or recordings of task-related activity). In such cases, investigators should account for variability from short data segments when estimating the power of statistical comparisons.

Since the firing rates and patterns of most GPe cells are variable, their firing characteristics may not be sufficient to categorize them. Most GPe neurons displayed HFD-P and LFD-B features at different times, but most of the time their firing pattern was undetermined (Fig. 6). Only a small proportion of GPe neurons fulfilled our stated criteria to be categorized as HFD-P or LFD-B (6.7 and 8%, respectively) for the entire record. Our results contrast with a previous report (Katabi et al., 2022), in which all GPe neurons were classified as either HFD-B (87%) or LFD-B (13%). While it is possible that the different results may be due to the limited sample size of our study (169 neurons in this study, 730 in the study of Katabi et al.), it is more likely that the differences arise from details of the methodology. For instance, our monkeys were not performing a task, unlike those in Katabi et al., which may have allowed GPe neurons to remain longer in the HFD-P or LFD-B state.

Our sample of GPe cells appears to come from a single, larger population of neurons, each capable of firing across a wide range of patterns and rates. Deister et al. (2013) reached a similar conclusion in their rodent studies (Deister et al., 2013). Other rodent studies also reported a wide variability and substantial overlap in various characteristics of firing, even among molecularly identified subpopulations of GPe cells (Mastro et al., 2014; Abdi et al., 2015; Hernandez et al., 2015; Abrahao and Lovinger, 2018; Cui et al., 2021; Ketzef and Silberberg, 2021). Some studies reported that GPe neurons within molecularly identified categories cover a more restricted range of firing characteristics (Abdi et al., 2015; Dodson et al., 2015; Cui et al., 2021). The cells that we recorded in monkeys may indeed belong to several genetic categories, whose specific firing patterns would only be evident if they could be classified with molecular markers. This approach is currently unavailable for studies in nonhuman primates.

The temporal fluctuations in firing rates, patterns, and functional states found in GPe neurons in healthy monkeys were also detected in parkinsonian monkeys, although the range of GPe neuronal firing rates and regularity was slightly narrower in the parkinsonian than in the healthy animals (Fig. 2*C*,*D*). Also, when analyzing the path taken by GPe neurons in the 3D space formed by firing rate, pause, and burst indexes, we found that GPe cells recorded in the healthy condition can take smaller minimal steps and larger maximal steps along their path, compared with parkinsonian cells (Fig. 5), further indicating a more restricted range of firing properties in parkinsonism. In addition, GPe neurons recorded in the parkinsonian condition spent less time in the HFD-P state than those recorded under healthy conditions, while the time spent as LFD-B increased. This difference may underlie previous reports of decreased firing rates of GPe neurons in the parkinsonian state (Miller and DeLong, 1987; Pan and Walters, 1988; Sterio et al., 1994).

It has been proposed that the heterogeneous firing rate of individual GPe neurons could reduce the tendency of GPe neurons to show synchronized firing (i.e., the variable firing would make it less likely for groups of cells to have correlated firing activities; Wilson, 2013), as happens in the healthy state. In the parkinsonian condition, the reduction in the range of firing rates could contribute to the increased synchrony among GPe neurons (Heimer et al., 2002). Our data do not allow a direct evaluation of this issue, as all units were recorded individually.

Several mechanisms could drive the constrained variability of GPe firing in the parkinsonian condition, from changes in ion channel composition (Chan et al., 2004; Chan et al., 2011), to alterations in local circuitry (Miguelez et al., 2012), to network effects (Schechtman et al., 2015). Furthermore, the reduction in the ranges of firing variability could reflect different effects of the parkinsonian state on specific subtypes of GPe neurons. Recent studies have shown that in parkinsonian mice, reduction in spontaneous activity is selective for GPe neurons that express the NPAS1 transcription factor, while parvalbumin (PV)-expressing neurons are minimally affected (Courtney et al., 2023). Further, synaptic inputs to GPe neurons are altered in parkinsonism, with weakened glutamatergic projections from the subthalamic nucleus (Pamukcu et al., 2020) and local collaterals among GPe neurons are strengthened (Miguelez et al., 2012). How these synaptic alterations affect the temporal variability remains to be determined.

There are several limitations to our study. It is possible that subtle shifts in the animal’s state of arousal contributed to the observed changes in firing patterns (Adler et al., 2010). However, spontaneous fluctuations in firing similar to those reported here in awake monkeys have also been identified in the GPe of anesthetized rats, and even in in vitro recordings (Deister et al., 2013), suggesting that the firing variations are a robust finding that is not fully explained by changes in the state of wakefulness. Also, our analysis of the relation of ECoG signals failed to show evidence for a strong link between the analyzed parameters of neuronal firing and the presence of strong low-frequency spectral power in the ECoG signals.

Another limitation is that the recordings in healthy monkeys were longer than those obtained in parkinsonian animals, reflecting the greater technical difficulty of successfully recording for long periods in parkinsonian monkeys. To account for this difference, we used the same number of ISIs (7,000) for all comparisons between data from the healthy and parkinsonian states.

Further, in contrast to rodent studies that can rely on transgenic mice to distinguish molecularly identified subsets of GPe neurons, we were not able to discriminate among genetically defined GPe neuron subtypes in our primate recordings. As mentioned above, studies in rodents have described two main groups of GPe neurons, those expressing PV and those expressing NPAS1 (Courtney et al., 2023), with additional markers to further subdivide these groups, potentially explaining some of the specific electrophysiological properties of these cells (Cui et al., 2021).

Finally, our data are based on single-unit recordings. Future work should include simultaneous recordings of multiple neurons to evaluate whether fluctuations of activity patterns are synchronized between GPe neurons.

Our results show that the firing patterns of GPe cells, both individually and as a population, span a wide range in both healthy and parkinsonian states, making clear distinctions between HFD-P and LFD-B cells difficult. Short recordings are insufficient for classification, and given the apparent heterogeneity of GPe activities, genetic markers (currently limited to rodents) are likely a better basis for classification than firing patterns alone.
